# Where is the Origin of the Last Normal Branch from Feeding Artery of Pulmonary Arteriovenous Malformations?

**DOI:** 10.1007/s00270-018-2063-4

**Published:** 2018-08-22

**Authors:** Miyuki Maruno, Hiro Kiyosue, Norio Hongo, Shunro Matsumoto, Hiromu Mori

**Affiliations:** Department of Radiology, Oita University Faculty of Medicine, 1-1 Idaigaoka Hasama-machi, Yufu-shi, Oita 879-5593 Japan

**Keywords:** Pulmonary arteriovenous malformations, Origin of the last normal branch, Reperfusion

## Abstract

**Purpose:**

Reperfusion via pulmonary-to-pulmonary arterial anastomoses is known as one type of recurrence of pulmonary arteriovenous malformations (PAVMs) after embolization. It is important to occlude the fistulous portion beyond the origin of the last normal branch from feeding artery of PAVMs to prevent recurrence. In this study, we evaluate the origin of the last normal branch by CT as well as its visibility on pulmonary arteriography (PAG).

**Materials and Methods:**

We reviewed forty patients with 77 PAVMs who underwent coil embolization between October 2007 and December 2017. All patients underwent MDCT before embolization. Axial and MPR CT lung images were reviewed with special interests in the origin of the last normal branch from feeding artery of PAVMs. The origin was classified into three portions, including sac, junction (portion just proximal to the sac) and proximal feeder (more than 5 mm proximal to the sac). We also evaluated whether PAG can depict the normal branches detected by MDCT.

**Results:**

MDCT showed that the last normal branch originated from sac in 30 PAVMs (39.0%), junction in 39 (50.6%), and proximal feeder in 8 (10.4%).On selective PAG, the last normal branch could be visualized in 30 PAVMs (39.0%), although it could not be visualized due to high-flow shunt in the other 47 PAVMs.

**Conclusions:**

Selective PAG frequently fails to demonstrate the last normal branch from feeding artery of PAVMs, which often originates from the sac. Pretherapeutic evaluation of CT images of the last normal branch is important to prevent reperfusion of PAVMs.

**Level of Evidence:**

Level 3, local non-random sample.

## Introduction

Pulmonary arteriovenous malformations (PAVMs) are rare vascular shunts communicating between pulmonary arteries and veins without an intervening capillary network. PAVMs may cause hypoxemia and serious complications such as embolic strokes, other paradoxical embolisms, brain abscesses, and massive hemoptysis [1–3]. Transcatheter embolization has been accepted as a standard treatment for PAVMs to remedy hypoxemia or to prevent such complications [1, 3–7]. However, recurrence of PAVMs after embolization can occur in up to 63% of cases [8]. Recurrent PAVMs also may cause serious complications, such as brain abscesses and paradoxical embolisms [5, 9]. Reperfusion via the anastomoses between the pulmonary arterial branches or systemic arteries and pulmonary arterial branches is known as one type of recurrence pattern after the transcatheter embolization of PAVMs [5, 9–11]. To prevent “reperfusion” via the anastomoses between pulmonary arterial branches, it is important to occlude the fistulous portion beyond the origin of the last normal branch from the feeding artery. The fistulous segment of the PAVMs usually shows saccular dilatation (sac), and it is generally thought that the last normal pulmonary arterial branch originates at the portion proximal to the sac. However, there is no report on the origin of “the last normal branch,” and we often experienced cases with the last normal branch originating from the sac in clinical practice of endovascular embolization. Furthermore, the last normal branch may not be identified on selective pulmonary angiography alone due to high-flow shunt. In this study, we retrospectively evaluated the origin of the last normal branch from the feeding artery of PAVMs by CT as well as its visibility on pulmonary angiography (PAG).

## Materials and Methods

### Patients

Between October 2007 and December 2017, 43 consecutive patients with PAVMs were treated by coil embolization. In all patients, multidetector CT (MDCT) was performed before embolization. Three cases of diffuse and tiny PAVMs along with cases that could not be evaluated due to motion artifact on CT were excluded. Consequently, 77 PAVMs in 40 patients were included in this study. There were 7 males and 33 females, with a mean age of 54 years (age range 14–85 years). The types of PAVMs were complex in one lesion and simple in the other 76 lesions, according to the classification by White et al. [4]. Ten patients were diagnosed with hereditary hemorrhagic telangiectasia according to the diagnostic criteria by Shovlin et al. [12].

### CT Image Techniques

MDCT scans were performed on 32-, 64-, or 320-channel MDCT scanners (Aquillion 32, CX, ONE, ONE Genesis, Toshiba, Tokyo, Japan). The following scan parameters were used: beam collimation 32 or 40 mm; pitch 27 or 65; rotation time 0.5 s; and 1.0 mm thickness at an interval of 1.0 mm. Images were acquired during deep inspiration of a single breath hold. All CT images used for evaluating pulmonary arterial branches were setting lung window to detect peripheral branches. Multiplanar reconstruction (MPR) images with an arbitrary cross section with lung window were also generated. Contrast materials were not used to evaluate pulmonary arterial branches because of a potential risk of air embolism during intravenous injection of contrast medium.

### Pulmonary Angiography (PAG) Techniques

All PAG images were obtained with a biplane flat-panel digital subtraction angiography (DSA) system (Infinix Celeve-I INFX-8000 V; Toshiba) using a femoral venous approach. Lobar PAG was performed via a 4F pigtail catheter positioned at the stem of the right or left pulmonary artery. First, 20–25 ml of contrast media (iopamidol 300 mg/dl; Iopamiron; Bayel Healthcare, Osaka, Japan) was injected at a flow rate of 7 ml/s by a power injection system. Selective PAG was then performed via a 4–5F catheter positioned at the segmental or subsegmental branch with 6–8 ml of contrast media by manual injection. Selective PAG images were obtained in frontal and oblique views to ensure optimal visualization of the feeding artery and aneurysmal sac.

### Evaluation Methods

**Fig. 1:**
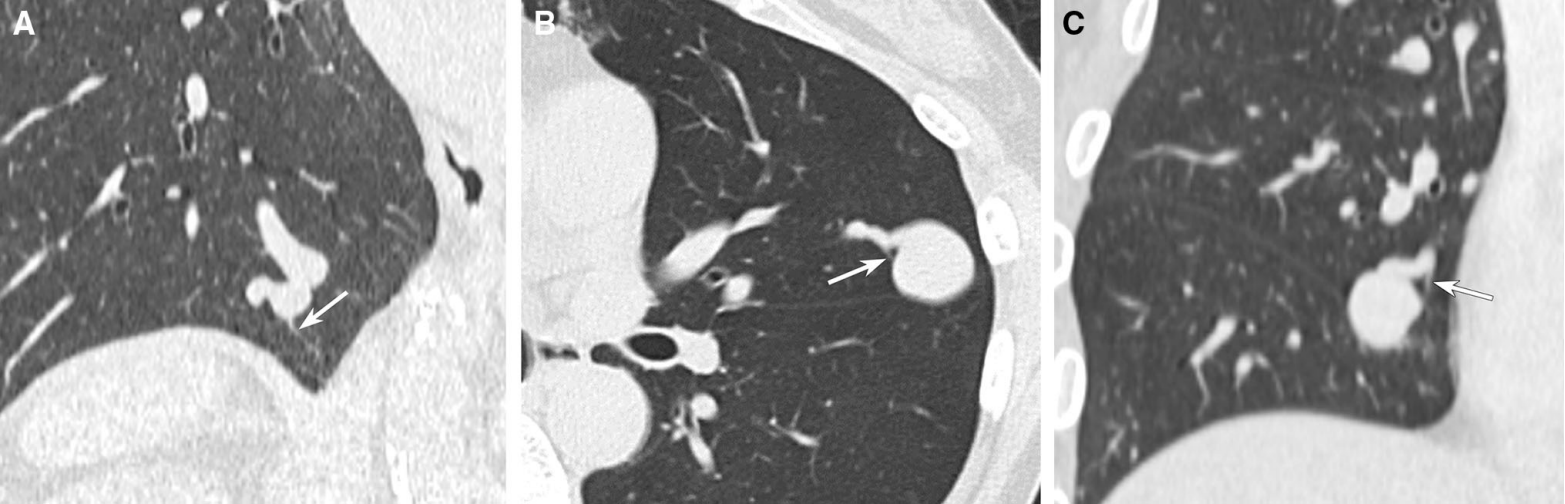
Types of origin of the last normal branch. MDCT images show three portions of the origin of the last normal branches. **A** The last normal branch originating from “sac”. Oblique coronal MPR CT image shows the last normal branch (arrow) originating from fistulous portion with saccular dilatation. **B** The last normal branch originating from “junction”. Axial CT image shows the last normal branch (arrow) originating from just proximal portion of saccular dilatation. **C** The last normal branch originating from “proximal feeder”. Oblique coronal MPR CT image shows the last normal branch (arrow) originating from the proximal feeder, more than 5 mm proximal to the saccular dilatation

**Fig. 2:**
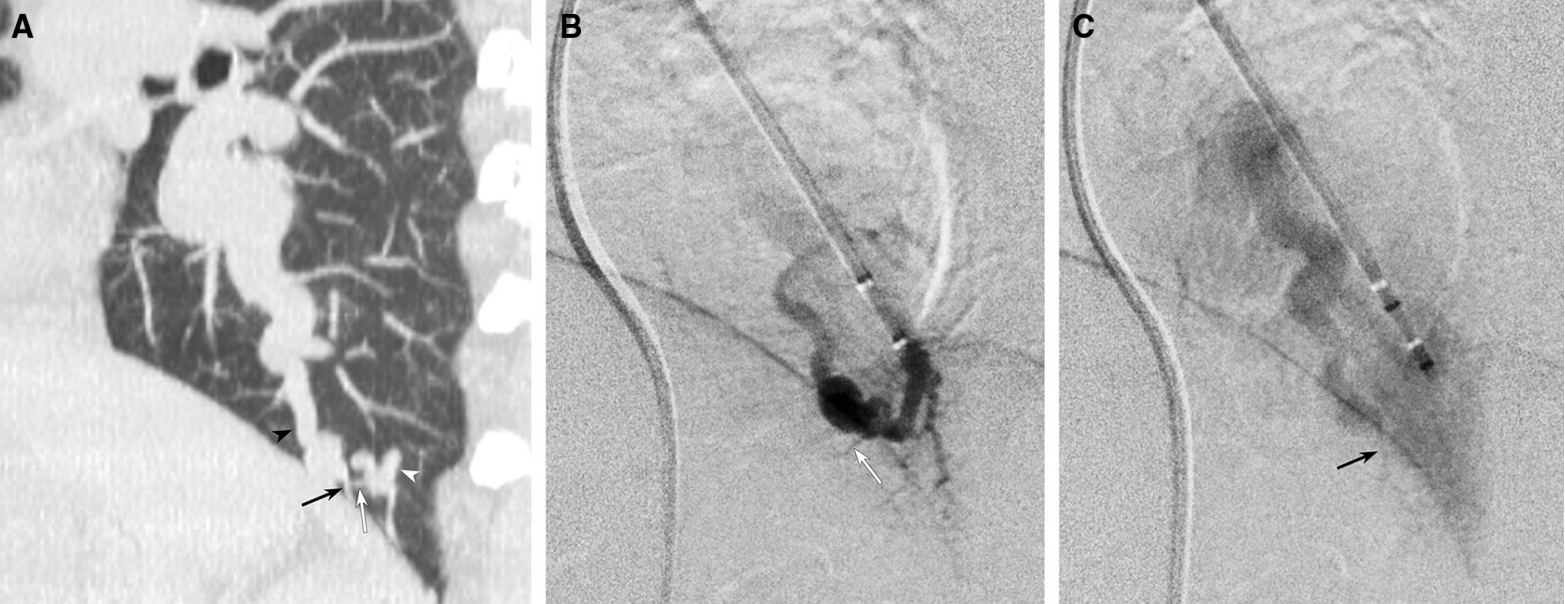
Definition of the last normal branch originating from the sac. **A** Oblique sagittal MPR CT image shows two branches originating from the sac. The direction of the proximal branch (white arrow) forms sharper angle to the feeding artery (white arrowhead) compared to the draining vein (black arrowhead). The distal branch (black arrow) forms very sharp angle with the draining vein (black arrowhead), but it forms obtuse angle to the feeding artery (white arrowhead). The proximal branch was classified as the last normal arterial branch, and the distal branch was classified as pulmonary vein due to the branching angle. **B** Lateral image of selective PAG during pulmonary arterial phase shows a pulmonary arterial branch (white arrow) from the sac which was detected on MDCT. **C** Lateral image of selective PAG during pulmonary venous phase shows a pulmonary venous branch into the sac (black arrow) which was detected on MDCT

Serial sectional images of axial and MPR CT with lung window were reviewed by two radiologists who had 14 and 28 years of experience (M. M. and H. K.). Special interest was given to the origin of the last normal branch from feeding artery of the PAVMs. Consensus was obtained in cases of discrepancy between both readers. The origin of the last normal branch was classified into three portions, including “sac,” “junction” (portion just proximal to the sac within 5 mm distance), and “proximal feeder” (more than 5 mm proximal to the sac) (Fig. 1). In case a branch originating from sac was detected, we defined the branch as a pulmonary arterial branch when the branch has a sharper angle to the feeding artery compared to the draining vein and/or does not distribute to territories of the adjacent pulmonary artery branches (Fig. 2).

Visibility of “the last normal branch” detected by MDCT was evaluated on lobar and selective PAG, respectively. PAG images were evaluated from frontal and oblique lateral views.

Characteristics of PAVMs, such as the size of the feeding artery and the maximum diameter of the sac, were also evaluated and compared between the PAVMs that the last normal branch was visualized (visible group) and was not visualized (no visible group) on selective PAG. Results were expressed as mean ± standard deviation. Continuous variables were compared parametrically using Student’s *t* test. A *p* value that was less than .05 was considered statistically significant.

## Results

### Evaluation of MDCT Images

MDCT images showed that the last normal branch originated from sac in 30 PAVMs (39.0%), junction in 39 PAVMs (50.6%), and proximal feeder in 8 PAVMs (10.4%) (Table 1).

**Table 1:** Origin of the last normal branch on MDCT

Origin of the last normal branch	Number of PAVMs
Sac	30/77 (39.0%)
Junction	39/77 (50.6%)
Proximal feeder	8/77 (10.4%)
Total	77/77

### Visibility of the Last Normal Branch on PAG

**Fig. 3:**
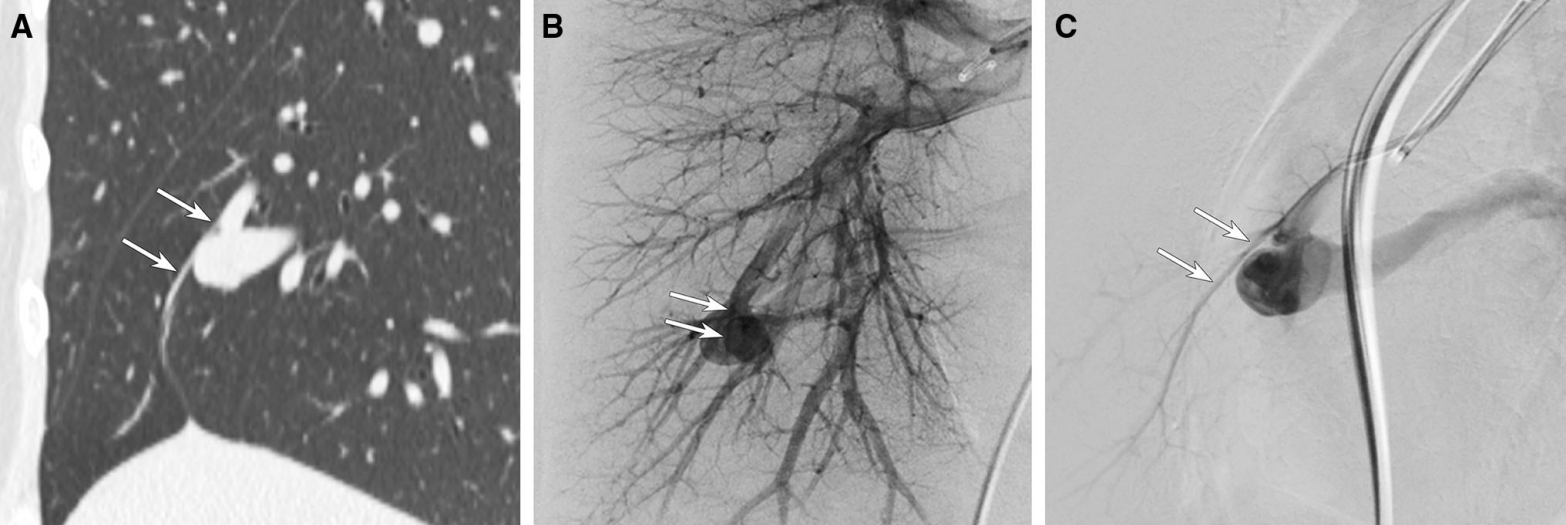
The last normal branch originating from junction visualized on both MDCT and PAG. **A** Sagittal MPR CT image shows the last normal branch (arrows) originating from junction. **B** Frontal image of lobar PAG can demonstrate the last normal branch (arrows) from junction detected on MDCT. **C** Lateral image of selective PAG can demonstrate the last normal branch (arrows)

Of all PAVMs, the last normal branch could be depicted in only 4 PAVMs (5.2%) on lobar PAG (Fig. 3B). A last normal branch from sac could not be depicted on lobar PAG (Table 2).

**Table 2:** Visibility of the last normal branch detected by MDCT on PAG

Origin of the last normal branch on MDCT	Visibility on PAG	
	Lobar PAG	Selective PAG
All portions (*n* = 77)	4/77 (5.2%)	30/77 (39.0%)
Sac (*n* = 30)	0/30 (0%)	12/30 (40%)
Junction (*n* = 39)	3/39 (7.7%)	15/39 (38.5%)
Proximal feeder (*n* = 8)	1/8 (12.5%)	3/8 (37.5%)

**Fig. 4:**
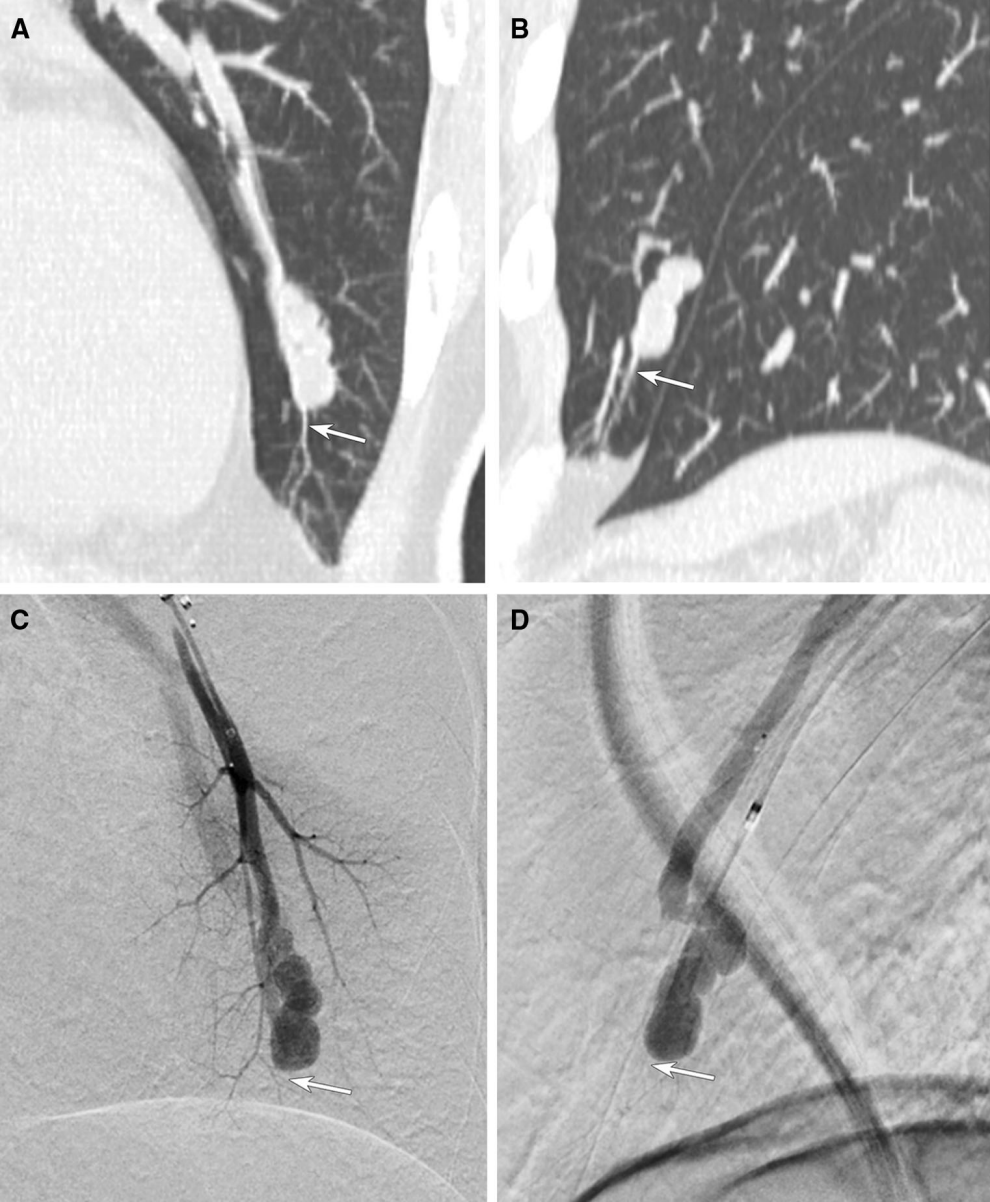
The last normal branch originating from sac cannot be visualized on selective PAG. **A**, **B** Oblique coronal (**A**) and sagittal (**B**) MPR CT images show the last normal branch (arrow) originating from sac of PAVM. **C**, **D** Frontal (**C**) and lateral (**D**) images of selective PAG cannot demonstrate the last normal branch detected on MDCT (arrow)

**Fig. 5:**
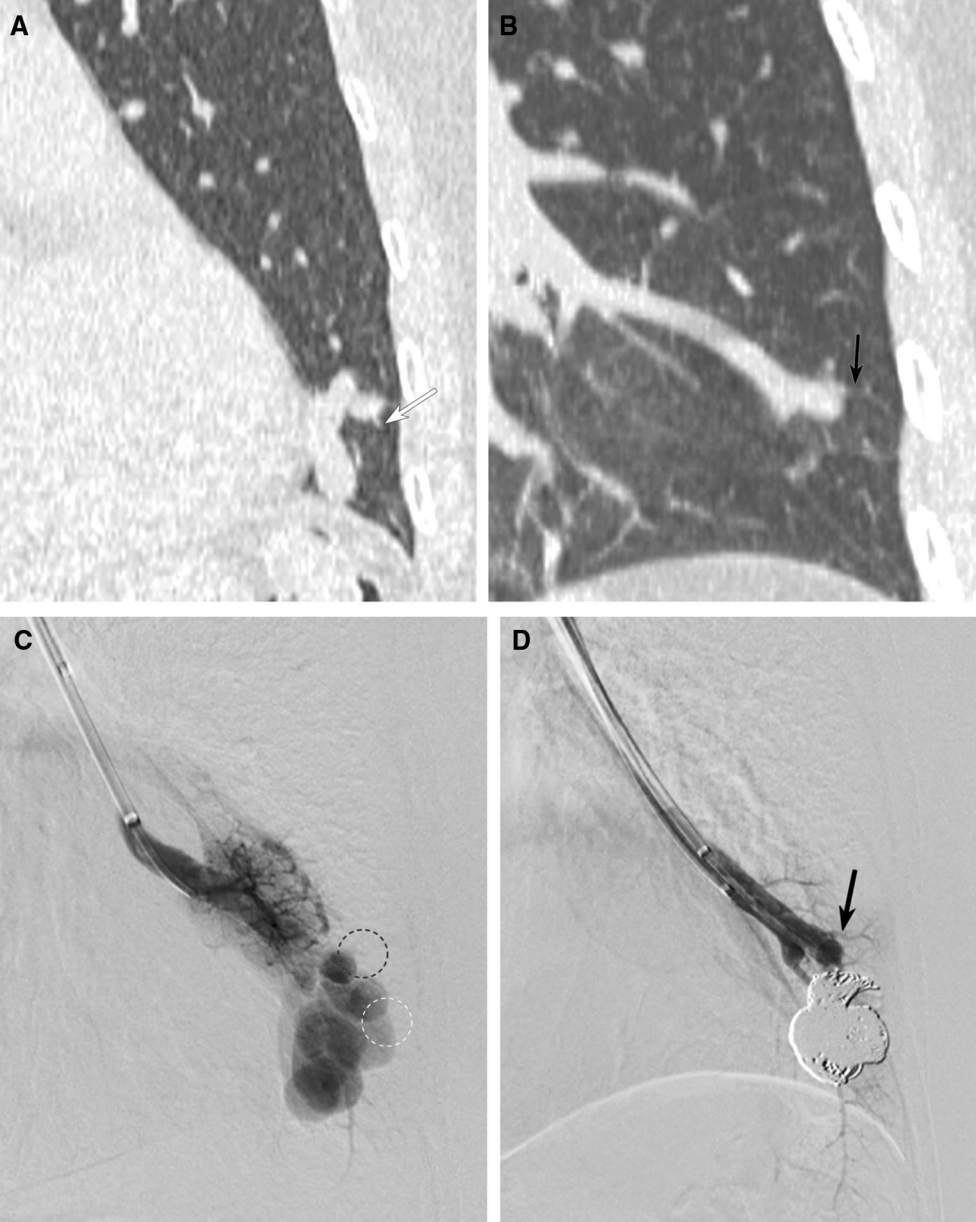
A case of PAVM in which selective PAG failed to demonstrate the last normal branch and proximal branches. **A** Oblique coronal MPR CT image shows the last normal branch originating from junction (white arrow). **B** Oblique coronal MPR CT image also shows the proximal branch from feeding artery (black arrow). **C** Frontal view of selective PAG cannot demonstrate both the last normal branch (circle of white dot-line) and proximal branch (circle of black dot-line) detected on MDCT. **D** Selective PAG after embolization clearly demonstrates the proximal branch from feeding artery (black arrow) which could not be visualized before embolization (circle of black dot-line in Fig. 4**B**)

The last normal branch could be visualized in 30 PAVMs (39%) on selective PAG. In the other 47 PAVMs (61%), it could not be visualized due to high-flow shunt (Figs. 4, 5). The visibility of the last normal branch in each origin was 40% (*n* = 12/30) from sac, 38.5% (*n* = 15/39) from junction, and 37.5% (*n* = 3/8) from proximal feeder. In 5 PAVMs, although selective PAG failed to demonstrate the last normal branch, microcoils were inserted into the last normal branch during embolization, and these branches could be detected by pretherapeutic MDCT.

### Characteristics of PAVMs

In all PAVMs, the mean diameter of the feeding artery was 7.24 ± 0.015 mm, and the mean maximum diameter of the sac was 19.10 ± 0.585 mm. The mean diameter of the feeding artery and the mean maximum diameter of the sac in the 30 PAVMs of visible group were 3.09 ± 0.11, 7.60 ± 0.77 mm, respectively. The mean diameter of the feeding artery and the mean maximum diameter of the sac in the 47 PAVMs of no visible group were 4.03 ± 2.08, 10.96 ± 12.39 mm, respectively. There is a significant difference in the size of the feeding artery and the sac of PAVMs between visible group and no visible group (*p* = 0.026, 0.005). Both the size of the feeding artery and the sac in the visible group were smaller than that in the no visible group.

## Discussion

**Fig. 6:**
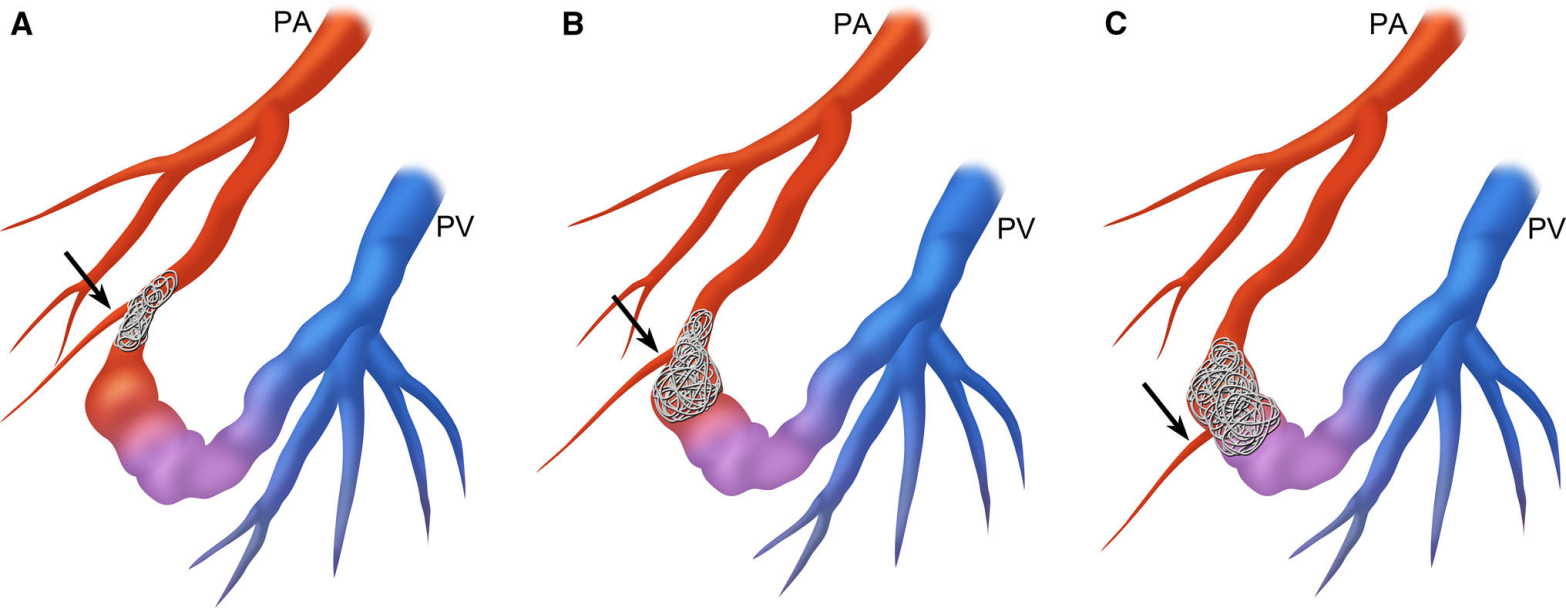
Schematic drawing of ideal embolization sites of the PAVM according to the origin of the last normal pulmonary arterial branch. **A** PAVM in which the last normal branch originates from proximal feeder. Coils are densely placed at the feeding artery close to the sac beyond the origin of the last normal branch (arrow). **B** PAVM in which the last normal branch originates from junction. Coils are densely placed from the proximal part of dilated fistulous portion (sac) beyond the origin of the last normal branch (arrow) to the feeder. **C** PAVM in which the last normal branch originates from sac. Coils are densely placed at the dilated fistulous portion (sac) beyond the origin of the last normal branch (arrow). PA: pulmonary artery, PV: pulmonary vein

Transcatheter embolization has been accepted as a standard treatment for PAVMs to prevent serious complications such as cerebral abscesses, paradoxical embolisms, and massive hemoptysis. Recently, a relatively high recanalization rate has been reported by using contrast enhanced CT [8] or time-resolved MR angiography [13]. When the blood flow of PAVMs remains after embolization, the risk of serious complications persists [5, 9]. The patterns of persistence of PAVMs after embolization are divided into three types, including recanalization through previously placed coils or plug, pulmonary-to-pulmonary artery reperfusion, and systemic-to-pulmonary artery reperfusion [5, 6, 9–11, 14]. The second embolization for recurrent PAVM is more difficult to treat compared to the initial embolization. Shimohira et al. [13] demonstrated 100% recurrence rate after the second embolization for recurrent PAVMs. Woodward et al. [10] reported pulmonary-to-pulmonary artery reperfusion was more difficult to re-treat successfully than recanalization. Therefore, it is important to embolize as not to recur at the initial embolization session. Recanalization can be theoretically prevented by dense occlusion with adequate embolic materials. Pulmonary-to-pulmonary artery reperfusion or systemic-to-pulmonary reperfusion is caused by recruitment of shunt flow via the potential anastomoses between the pulmonary arterial branches, or the pulmonary artery and the systemic arterial branches such as bronchial artery. Therefore, it is important to occlude the fistulous portion beyond the origin of the last normal branch from feeding artery of PAVMs to prevent pulmonary-to-pulmonary artery reperfusion (Fig. 6). Some authors have noted the importance of embolization of feeding artery close to the sac, while others demonstrated the efficacy of venous sac embolization to prevent persistence [9, 11, 15–17]. However, they did not mention where the fistulous point in PAVM is. Furthermore, coil embolization of a large sac requires a large number of coils. It is not necessary to occlude the sac with a large number of coils for cases in which the last normal branch originates from the proximal feeding artery. In contrast, sac embolization as dense as possible is required for cases in which the last normal branch originates from the sac in order to prevent reperfusion. If the sac is embolized roughly and reperfusion via the anastomosis between the pulmonary arterial branches to the sac occurs, it is extremely difficult to embolize fistulous point via the pulmonary artery for such cases. Since the feeding artery is densely embolized, it is difficult to advance the microcatheter to the fistulous point beyond the coil mass in the feeding artery. Therefore, as dense packing as possible of the sac is required for the PAVMs in which the last normal branch originates from the sac, even if costly. Regarding the suitable embolic materials either coils or plugs, plugs may be suitable for the cases of the last normal branch originating from the proximal feeding artery without tortuous access route to the fistulous point. We do not recommend the use of vascular plugs for the cases that the last normal branch originates from the sac or junction. Identification of the origin of the last normal branch from the feeding artery of PAVMs is necessary to decide the occlusion point, and selection of devices based on this anatomy for each case is important for reasonable and successful occlusion of PAVMs.

As mentioned before, it has been generally thought that the last normal branch originates from the proximal feeding artery before the saccular dilatation. This study showed that the last normal branch originates from the sac or junction in the majority of PAVMs (89.6%). Furthermore, “the last normal branch” could not be identified in 61% of PAVMs on selective PAG due to high-flow shunt. High-flow AVM may cause hypoperfusion in surrounding normal tissue, known as “steal phenomenon”. Therefore, the selective PAG, as shown in Fig. 5, fails to demonstrate the last normal branch as well as the proximal branches from feeding artery due to high-flow shunt. These pulmonary arterial branches become visible on PAG after selective occlusion of the fistulous portion of PAVMs. MDCT in advance clearly depicts the normal branches that have been obscured by the high-flow shunt. These results suggest that the sites of embolization of PAVMs determined by angiographic findings alone are too proximal for ideal embolization in many cases. Because the selective PAG was performed via manual injection, the low flow rate of contrast materials during PAG with manual injection may be a one of the factors for low visibility of the normal branch. Actually, the size of PAVMs where the last normal branch was not visualized on selective PAG was significantly larger than that of PAVMs where the last normal branch was visualized. Therefore, the pretherapeutic assessment of the MDCT findings of the last normal pulmonary artery branch is important for embolization of PAVMs. Selective angiography under flow control of feeding artery with balloon catheter can reduce the shunt flow, and therefore, it may improve the visibility of the last normal branch of PAVMs.

There are some limitations in this study. The sample size is relatively small, and the study design is retrospective and based on a single center. These factors may affect the bias control. Further prospective studies with a larger number of subjects are necessary. The image on MDCT was static, and therefore, the identified last normal branches could not be completely distinguished from other structures connecting the PAVMs, such as the inflammatory scar.

In conclusion, the last normal branch from the feeding artery frequently originated from the sac or junction of PAVMs, which is often invisible on selective PAG. Pretherapeutic evaluation of MDCT images with special interest in the origin of the last normal branch from the feeding artery is mandatory to decide the occlusion segment.

## Abbreviations

DSA: Digital subtraction angiography
MDCT: Multidetector CT
MPR: Multiplanar reconstruction
PA: Pulmonary artery
PAG: Pulmonary angiography
PAVM: Pulmonary arteriovenous malformation
PV: Pulmonary vein
